# Bibliography

**Published:** 1904-06

**Authors:** 


### Bibliography.

---

Irregularities of the Teeth and their Treatment. By Eugene S. Talbot, M.S., D.D.S., M.D., LL.D., Professor of Stomatology, Illinois Medical College; Honorary President of the Dental Section of the Tenth International Medical Congress, Berlin, 1890, etc., etc. Fifth Edition. Five hundred and eighty-one Illustrations. The S. S. White Dental Manufacturing Company, Philadelphia, 1903.

This work of Dr. Talbot was very thoroughly reviewed in a former edition, and it is, therefore, not possible and perhaps unnecessary to enlarge upon the previous judgment. It comes to us in this edition without any change from that of the fourth; the text, paging, and binding are in this followed with scrupulous exactness. The author gives the reason for this in the fact that "Since the fourth edition of this work appeared, practically no modifications of the general principles therein outlined have been made. The laws by which deformities of the jaws and irregularities of the teeth are governed had been worked out on broad scientific lines." The author, however, adds an "Introduction," not in former editions, in order to "outline . . . the working of the developmental principles governing heredity and environment as to facilitate their application to the topics discussed in the different divisions of the subject."

The author in this assumes that the human face is gradually deteriorating, for he states: "The human face, with all its beauty, considered from the stand-point of food-getting, chewing, and combat is, as Minot has shown, assuming an embryonic type. The jaws are needed less and less for the purpose mentioned, hence, under the law of economy of growth, the resultant disuse sacrifices them for the benefit of the growing brain and nervous system and the dermal elements of the skull." This assertion is made, it seems to the writer, without sufficient proof to give it support. It is a recognized fact that at no period in the history of the human race has there been more evidence given of brain activity and consequently brain development, than during the nineteenth century, and, judging the race *en masse*, there certainly is no marked evidence of jaw deterioration for "the benefit of the growing brain and nervous system and the dermal elements of the skull." It is thought, therefore, that the Introduction, while it may briefly cover the thoughts of the author, does not strengthen the edition. This may be a matter for regret, for the author, more than any other writer, has broadened the scientific view of irregularities of the jaws and surrounding parts. He has taught the professional mind to probe deeper into the mysteries of development and has shown that if we expect to comprehend irregular presentations we must understand the changes that have gradually, through generations, led up to this. Hence this work has forced its way through four editions, necessitating a fifth to meet the demand.

To perform a practical piece of work in the regulation of teeth may seem to the average dental mind simply as a mechanical problem. If success follows this superficial idea it must be regarded as accidental. All treatment of abnormal conditions must be based on a thorough understanding of the processes that have led up to the deterioration. If this be not done then treatment becomes wholly empirical.

It is to the credit of leading writers on orthodontia that this important foundational idea has not been lost in the effort to appeal to the practical man. Dr. Talbot has, in all probability, accomplished more than others in this direction, in that he has worked out his problems upon lines of development and carried these logically to the given result.

It is thought the author has been wise in not making any change in this edition. The, so-called, practical man will object to the limited space devoted to appliances and methods of use. This remains the same as in the fourth edition. The value of this work cannot be based on this, but upon the research given and preceding this, to make orthodontia something more than a series of screws, springs, and bands, but problems that require for their solution a knowledge of changes produced by heredity, evolution, and environment, and when these are understood the difficulties may be scientifically conquered. To this end our author has marked a path for all to follow. The book should be in the library of every practitioner of this very difficult branch of dental practice.

Irregularities of the Teeth and their Correction. By J. N. Farrar, M.D., D.D.S.

Dr. Farrar has for a long time been considering the advisability of publishing an edition of his work, "Irregularities of the Teeth and their Correction," in smaller volumes, to be sold at two dollars and fifty cents per volume, so that the younger members of the dental profession may obtain a volume treating upon any particular case desired, at a less price than the larger volumes. But by dividing each of the large volumes (published) into three small volumes, and the third large volume (which is in process to be published) is added, they all would make nine volumes, which, at two dollars and fifty cents per volume, would amount to twenty-two dollars and fifty cents for the full set, in cloth binding, which would be four dollars and fifty cents more than his former price.

As there would be an extra expense in making so many small volumes, Dr. Farrar has finally decided that instead of issuing his work in smaller volumes, it would be better for the dental profession, as well as all other parties interested, to lessen the usual prices by curtailing some of the sale expenses, and making some personal reductions, thereby permitting a material reduction. For illustration, a volume in cloth would be only four dollars instead of six dollars, the usual price, and for the higher grade of binding the same reduction of two dollars per volume would be made. At this rate per volume, in cloth binding, "Irregularities of the Teeth and their Correction," when all are published, will be obtainable at the remarkably low price of twelve dollars for the complete work of three volumes, of which two volumes are now published and the third is being prepared for the publisher; being pushed as fast as the author's leisure time will permit of the proof-reading. Dr. Farrar's manifold professional labors do not permit him to progress as rapidly as we would like.

Sunshine Thoughts for Gloomy Hours: Prose and Verse. By Geo. H. Chance, D.D.S., M.D., J. K. Gill Company, Publishers, Portland, Ore.

It seems not a very long time in the history of this country when all that the East knew of Oregon was from the travels and descriptions of intrepid explorers who braved exposures, Indian raids, and months of travel overland that curious readers might be made familiar with this far away western border of our Continent.

The writer's boyish imagination revelled in the thought of the time when he possibly might investigate personally this new life as it was then pictured; but that time never came, and now cities and towns have made the Pacific Coast so populous that no longer is there any wild country there or between the great oceans, and at the base of Mount Tacoma and Ranier we find the same æsthetic tastes as in the older settled regions.

Now comes our old friend Chance, of Portland, Ore., with the sunshine of his thoughts,—and who that has ever been an hour in his society has not felt their warming and enlivening influence. Dr. D. Solis Cohen, in his "Introduction," says that "the optimist is one of the world's treasures," and this he applies very truly to the author of this book of "Sunshine Thoughts."

The latter divides the book into articles of prose and verse. Treating themes "Patriotic," and again he discourses on "Fads, Facts, and Fancies;" then becomes "Fraternal," and enters into the domain of Freemasonry. Here the author seems much at home, as he writes himself as a 33°; also, as an "Odd Fellow," he grows enthusiastic over the good deeds of that Order; and finally he descends from Parnassian heights to measure his muse with his prosaic profession.

The near approach of the Fourth International Dental Congress at St. Louis seems to make appropriate the following extract from the address the author delivered at the opening of the Pacific Coast Dental Congress in 1897.

"And when this congress stands adjourned,
May those from near and far
Reach each his home in blissful mood,
Without a pain or jar.
But, when adjourned, be not in haste
To reach your Eastern homes,
Till you have seen the Cascade Range,
Its snow-capped peaks and domes."

The altruistic thought permeating these effusions of our author will be apparent in every line, but especially so in the last "International Anthem" to "England and America," which seems to the writer to be the best in the collection. The author has evidently lived to carry sunshine everywhere, and those who have known him best can well understand that these poems simply typify the soul of the man enshrined in words.

